# Kidney organoids generated from erythroid progenitors cells of patients with autosomal dominant polycystic kidney disease

**DOI:** 10.1371/journal.pone.0252156

**Published:** 2021-08-02

**Authors:** Roberta Facioli, Fernando Henrique Lojudice, Ana Carolina Anauate, Edgar Maquigussa, José Luiz Nishiura, Ita Pfeferman Heilberg, Mari Cleide Sogayar, Mirian Aparecida Boim

**Affiliations:** 1 Nephrology Division–Federal University of São Paulo, São Paulo, Brazil; 2 Cell and Molecular Therapy Center (NUCEL), School of Medicine, University of São Paulo, São Paulo, SP, Brazil; 3 Biochemistry Department, Chemistry Institute, University of São Paulo, São Paulo, SP, Brazil; Center for Molecular Biotechnology, ITALY

## Abstract

**Background:**

Kidney organoids have been broadly obtained from commercially available induced pluripotent stem cells (iPSCs); however, it has been a great challenge to efficiently produce renal organoid models from patients with autosomal dominant polycystic kidney disease (ADPKD) that recapitulate both embryogenesis and the mechanisms of cystogenesis.

**Methods:**

Blood erythroid progenitors (EPs) from two ADPKD patients and one healthy donor (HC) was used as a comparative control to normalize the many technical steps for reprogramming EPs and for the organoids generation. EPs were reprogrammed by an episomal vector into iPSCs, which were differentiated into renal tubular organoids and then stimulated by forskolin to induce cysts formation.

**Results:**

iPSCs derived from EPs exhibited all characteristics of pluripotency and were able to differentiate into all three germ layers. 3D tubular organoids were generated from single cells after 28 days in Matrigel. HC and ADPKD organoids did not spontaneously form cysts, but upon forskolin stimulation, cysts-like structures were observed in the ADPKD organoids but not in the HC-derived organoids.

**Conclusion:**

The findings of this study showed that kidney organoids were successfully generated from the blood EP cells of ADPKD patients and a healthy control donor. This approach should contribute as a powerful tool for embryonic kidney development model, which is able to recapitulate the very early pathophysiological mechanisms involved in cytogenesis.

## Introduction

Autosomal dominant polycystic kidney disease (ADPKD) is the most frequent genetic cause of chronic kidney disease (CKD) worldwide, and it is one of the most prevalent inherited monogenic disorders, affecting a population estimated at 1:400–1000 [1]. ADPKD is caused by mutations in the PKD1 and PKD2 genes, coding for polycystin-1 (PC1) and polycystin-2 (PC2), respectively, resulting in cilia alterations and cyst formation. Both proteins modulate numerous molecular pathways as well as tissue morphogenesis and function. However, the exact molecular mechanisms underlying cystogenesis remain unclear. It has been well accepted that a germline mutation followed by a somatic mutation (second-hit) in the normal allele is necessary for cystogenesis [2, 3]. Moreover, based on results from orthologous mouse models, it has been demonstrated that an additional renal insult (third-hit) is required for rapid and severe cyst growth in mature kidneys [4, 5]. It seems that in humans, most second hits occur during renal development, leading to cystic formation and growth even *in utero*. Therefore, the severity and the rate of progression of kidney disease resulting from PKD1 and PKD2 mutations may be influenced by several factors, including the timing of inactivation of the genes, kidney developmental stage, environmental influence, presence of concomitant renal lesions, and diversity of genetic mutations.

Although there has been innumerable data concerning the pathophysiological mechanisms of ADPKD collected from studies employing orthologous and nonorthologous mouse models, only a handful of studies focusing on human cellular models of cystogenesis are available [6]. In 2007, Takahashi et al. [7] successfully reprogrammed somatic cells into pluripotent stem cells with embryonic properties, naming them “induced pluripotent stem cells” (iPSCs). Since then, many somatic cell sources have been reprogrammed into iPSCs from either animal or human tissues (hiPSCs) [7], with adult fibroblasts representing the main source of the latter. In 2013, Freedman et al. [8] induced hiPSCs from the fibroblasts of ADPKD patients and found reduced ciliary expression of polycystin-2, supporting the use of such cells to investigate polycystic disease (PKD) pathophysiology. More recently, Chen et al reported in 2016 that they had obtained hiPS cells from erythroid progenitor cells [9].

Over the last few years, there have been significant advances in the generation of 3-dimensional organoids from iPSCs [7, 10–12]. Primary kidney tubular epithelial organoids can be derived from kidney tissue, as well as from urine, and these organoids are an important tool for personalized disease modeling, as shown by Schutgens et al. [13]. In subsequent studies, Freedman et al. [14] were able to generate kidney organoids derived from a commercially available hiPS cell line, establishing a protocol to generate the cell types present in the metanephric mass that give rise to all structures of the nephron. Then, by knocking down the PKD1 or PKD2 genes, these investigators observed cyst formation from kidney tubules. In view of all these advanced and innovative technologies, we aimed to obtain iPSCs from the circulating erythroid progenitor cells of ADPKD patients and test the development of kidney organoids *in vitro* to recapitulate the steps involved in organogenesis, trying to evoke some pathophysiological events related to early cystogenesis.

## Methods

### Patient selection

Blood samples were collected from two unrelated female patients (PT1 and PT2) clinically diagnosed with ADPKD (PT1: 66 years old, eGFR 43.8 mL/min per 1.73 m2 and PT2: 56 years old, eGFR 60.2 mL/min per 1.73 m2), followed up at the Polycystic Kidney Disease Outpatient Clinic of the Nephrology Division of the Federal University of São Paulo (UNIFESP), and another sample was collected from one healthy female, which served as a control to stablish and normalize the technique. The ADPKD diagnosis was based on a positive family history and on the presence of renal cysts, according to the ultrasonographic diagnostic criteria by Pei et al. [15]. (Family pedigrees are presented in S1A Fig). Both patients had cystic kidneys and livers (Magnetic Resonance Images are presented in S1B Fig). The study was reviewed and approved by the Ethics Advisory Committee of the University (CEP UNIFESP, “Comitê de Ética em Pesquisa da Universidade Federal de São Paulo”, Nr.1199.-0079-10/2018). After an interview to explain the purpose of the study, both patients and the healthy control signed the informed consent form.

### Expansion of erythroid progenitor cells isolated from whole blood

Blood samples were collected in a vacutainer tube containing EDTA (as an anticoagulant) at room temperature. Erythroid progenitor (EP) cells were then separated through a RosetteSep method (15216; Stem Cell Technologies), which removed unwanted cells, such as mature RBCs and platelets, while retaining the desired EP population. Due to the low concentration of EPs in whole blood, the cells were expanded and cultured in a specific culture medium containing cytokines and supplements (X-vivo medium, Lonza). EP cells were identified by flow cytometry using the transferrin receptor (CD71) and glycophorin A (GlyA) as markers labeled with GFP (S2 Fig).

### Reprogramming erythroid progenitor cells

Expanded EP cells were collected and nucleofected using an Epi5 Episomal iPSC Reprogramming kit (Thermo Fisher) containing an optimized mixture of five reprogramming factors (Oct-4, Sox2, Lin28, L-Myc and Klf4). Transfection (electroporation) was performed with episomal vectors (virus free, nonintegrating) from a Lonza Nucleofactor kit. After transfection, cells were plated in ReproTeSR-specific culture medium for reprogramming (Stem Cell Technologies) under standard cell culture conditions (37°C, 95% CO_2_, and 5% O_2_).

### Karyotyping

To verify whether the reprogramming process preserved chromosomal integrity, a karyotype analysis was carried out at the Genetic Department of UNIFESP. Cells were treated with 100 μl of colchicine (0.08 μg/ml) in 5 ml of iPS cell culture medium to induce cell cycle arrest, which was followed by the addition of a 0.075 M hypotonic KCl solution to induce nuclear swelling; cells were then fixed with a mixture of methanol and acetic acid (3:1). Metaphase chromosomes were harvested, and Wright staining was used for cytogenic analysis of G-bands (S3 Fig).

### iPSC colony characterization

**Fig 1:**
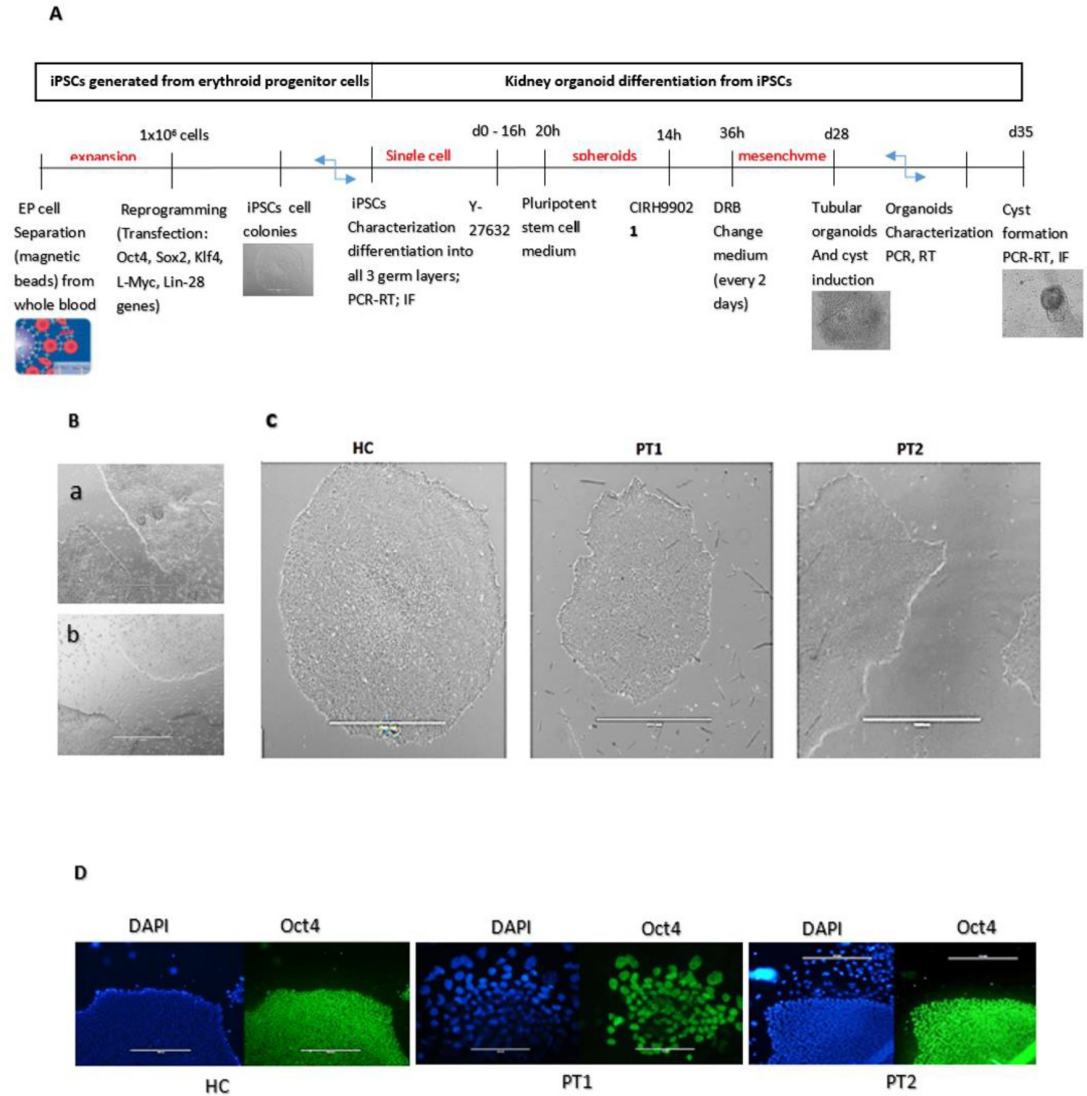
Generation and characterization of iPSCs. iPSCs were reprogrammed from expanded erythroid progenitor (EP) cells from a healthy control (HC) and two different ADPKD patients (PT1 and PT2). A. Schematic stepwise generation of kidney organoids B. Similarity of iPSC (a. typical image of iPSC from the commercial H9 embryonic cells. b. iPSC from progenitor erythroid cells from the healthy control) C. Morphology of iPSC colonies D. Characterization of iPSC pluripotency observed under an EVOS fl inverted digital fluorescence microscope. OCT4 antibody labeling was used as a pluripotency marker to characterize the pluripotency nature of the HC, PT1 and PT2 colonies. Cell nuclei were labeled with DAPI (scale bar for Fig 1B: 200 μm; scale bar for Fig 1C: 1000 μm; scale bar HC / PT2 for Fig 1D:1000 μm; scale bar PT1 for Fig 1D: 200μm).

EP cells were reprogrammed by epissomal vectors formed typical iPSC colonies and presented a normal karyotype (S3 Fig). The iPSC colony characteristics were compared with those of an established control derived from the H9 line cell (hESC, Human Embryonic Stem Cells, WA09, available from the Wicell Research Institute, USA) (Fig 1B). Since the iPSCs presented typical morphology, the pluripotency markers were identified by immunofluorescence. Cells were fixed with 4% paraformaldehyde for 30min at room temperature. After fixing, samples were washed with PBS three times, blocked in 5% PSA and 0.3% Triton-X-100/DPBS, and incubated overnight with primary antibodies (Cell Signaling, Ma, EUA), namely, OCT4 (1:400 dilution) as a test of pluripotency. On the next day, a new slide was prepared, washed in PBS and incubated with an Alexa Fluor secondary antibody (1:200 dilution, Cell Signaling), and DAPI (1:1000, Invitrogen, MA, USA) was added for nuclear identification.

### Capacity of iPSCs to differentiate into all three germ layers

iPSCs were grown in 6-well plates coated with Matrigel (1:10, BD Biosciences, NY, EUA) in mTeSR1 culture medium (Stem Cell Technologies, BC, Canada). To prevent the iPSCs from differentiating, the colonies were passaged manually by mechanical fragmentation with a pipet tip under a microscope, or they were dissociated by enzymatic digestion using Gentle Cell (Life Technologies) (S4 Fig). Using these methods, iPSCs were maintained for 22 serial passages to ensure that cells properly incorporated the reprogramming factors. The iPSCs were then stimulated to differentiate into the three germ layers using a STEMdiff Trilineage Differentiation kit (Stem Cell Technologies). After five days, cells were harvested for analysis of lineage-specific markers of mesoderm and endoderm, and after seven days they were analyzed for ectoderm, according to the STEMdiff Trilineage Differentiation protocol (S5 Fig).

### Molecular characterization of all three germ lines by qRT-PCR

Total RNA from cultures of the three germ layers was extracted using a GE RNA Spin Mini kit (GE Healthcare, USA), and then converted into cDNA using a High Capacity Reverse Transcription Kit (Applied Biosystems, USA) according to the manufacturer’s instructions. Quantitative real-time PCR (qRT-PCR) was carried out using a SYBR Green PCR Kit (Applied Biosystems, USA) according to the manufacturer’s protocol. mRNA expression levels were calculated using the 2^ΔΔ^ Ct method. Hydroxymethylbilane synthase (HMBS) and glyceraldehyde 3-phosphate dehydrogenase (GAPDH) were used as internal controls, and the expression of OCT4 was used as a quantitative control. The expression of SOX17 was used to identify differentiation into endoderm, CXCR4 was used to identify differentiation into mesoderm, and PAX6 was used to identify differentiation into ectoderm (S6 Fig). All primer sequences are specified in S1 Table, and the efficiency curve was evaluated for each primer pair.

### Immunofluorescence for the different germ layers

iPSCs were induced to differentiate on coverslips in six-well plates. After differentiation, the coverslips were removed from the original wells and transferred to a new plate, where the cells were fixed with 4% paraformaldehyde and then washed with PBS, as described above. Primary antibodies against OCT4 (1:400; Cell Signaling), CXCR4 (1;100; Cell Signaling), PAX6 (1:100; Thermo Fisher), and SOX17 (1:100; R&D Systems) were used. Fluorescence was assessed using an Alexa Fluor secondary antibody (1:200; Cell Signaling).

### Kidney organoid differentiation from iPSCs

Renal organoids were generated following the protocol described by Cruz et al. [6] with some adaptations. Briefly, 50,000 iPSCs from the HC and two ADPKD patients were used to produce suspensions of single cells with Accutase detachment solution (Stem Cell Technologies). Single cells were supplemented with 10 μM Rho-kinase inhibitor (Y27632, Stem Cell Technologies or Stemgent). After 16 h, the culture medium was replaced with 1 mL of mTeSR1 + 2.5% Matrigel + 10 μM Y27632. After 20 h, the medium was replaced with 1 mL of mTeSR1 only; 14 h later, the culture medium was replaced, and cells were induced with 6 μM CHIR99021 (AT104; Stem Cell or Stemgent GSK-3b inhibitor/Wnt pathway agonist) + Advanced RPMI (Thermo Fisher) + Glutamax (Life Technologies) + 10 μg/ml antibiotic (Ampicillin; 1:1000; Gibco). Finally, 36 h later, the culture medium was replaced with DRB medium containing Advanced RPMI (Thermo Fischer) + Glutamax (Thermo Fischer) + B27 Supplement (17504–044; Life Technologies) + antibiotic. The DRB medium was changed every three days for 28 days. This period (28 days) was enough for the organoids to be visible by microscopy.

### Molecular characterization of organoids by RT-PCR

Semi-quantitative gene expression was evaluated by RT-PCR during the embryogenic process with total RNA being purified from the cell structures formed at days 0 (iPSCs) and at 14, 21, 28, and 35 days after differentiation induction. The expression of WT1 (developing kidney), AQP1 (proximal tube), and ECAD (cyst epithelium marker) was evaluated. GAPDH was used as an internal control. The expression of OCT4 was used as a quantitative standard.

### Structural characterization of organoids assessed by immunofluorescence

The specific structure of the organoids was identified by immunofluorescence using antibodies against the following proteins: Nephrin 2 (NPHS2, 1:100 dilution; Abcam, UK) for podocytes, AQP1 (1:500 dilution; Sigma; Munich, Germany) and NHE3 (1:500 dilution; Abcam) for proximal tubules, AQP2 (1:100 dilution; Abcam) for distal tubules and ECAD for cystic structures (1:500 dilution R&D Systems). Fluorescent images were captured using an EVO microscope from Thermo Fisher. Positive and negative controls for all antibodies are shown in S7 Fig.

### Cyst induction

Forskolin (10 μM; Sigma), an adenyl cyclase inhibitor, was used to induce cyst formation. Forskolin was added to adherent cultures on the 28th day of differentiation, since this period was coincident with the onset of tubular protein expression.

## Results

### Obtaining iPSCs from erythroid progenitors (EPs)

The protocol employed to obtain kidney organoids from erythroid progenitors is summarized in suA. EPs expansion resulted in 1x10^6^ cells after approximately 20 days (S2A Fig), and approximately two months later, EPs were identified by their expression of the markers CD71 and GlyA. The efficiency of the separation method revealed expression; 88% of the cells were CD71 positive, and 57,4% were GlyA positive (S2B Fig).

After verifying that the cells were EPs, they were transfected with reprogramming factors (Oct-4, Sox2, Lin28, L-Myc and klf4) and then were maintained in a specific medium for reprogramming (ReproTeSR, Stem Cell Technologies) for approximately five days. iPSCs developed typical cellular characteristics by forming colonies with a morphology distinct from that of non-reprogrammed cells present in the same cell culture. Cells were monitored until we observed typical iPSC-like colonies that were similar to the colonies of commercial H9 cell line (Fig 1B). iPSC colonies were also recognized by their relatively larger size, tight cell packing, well-defined homogeneous shape and regular edges. Identification was facilitated by the low degree of colony overgrowth from differentiated cells in feeder-free reprogramming medium (Fig 1C). No detectable differences were found among iPSC colonies from the HC and ADPKD patients (PT1 and PT2) in terms of colony morphology and pluripotency markers, as shown in Fig 1D.

**Fig 2:**
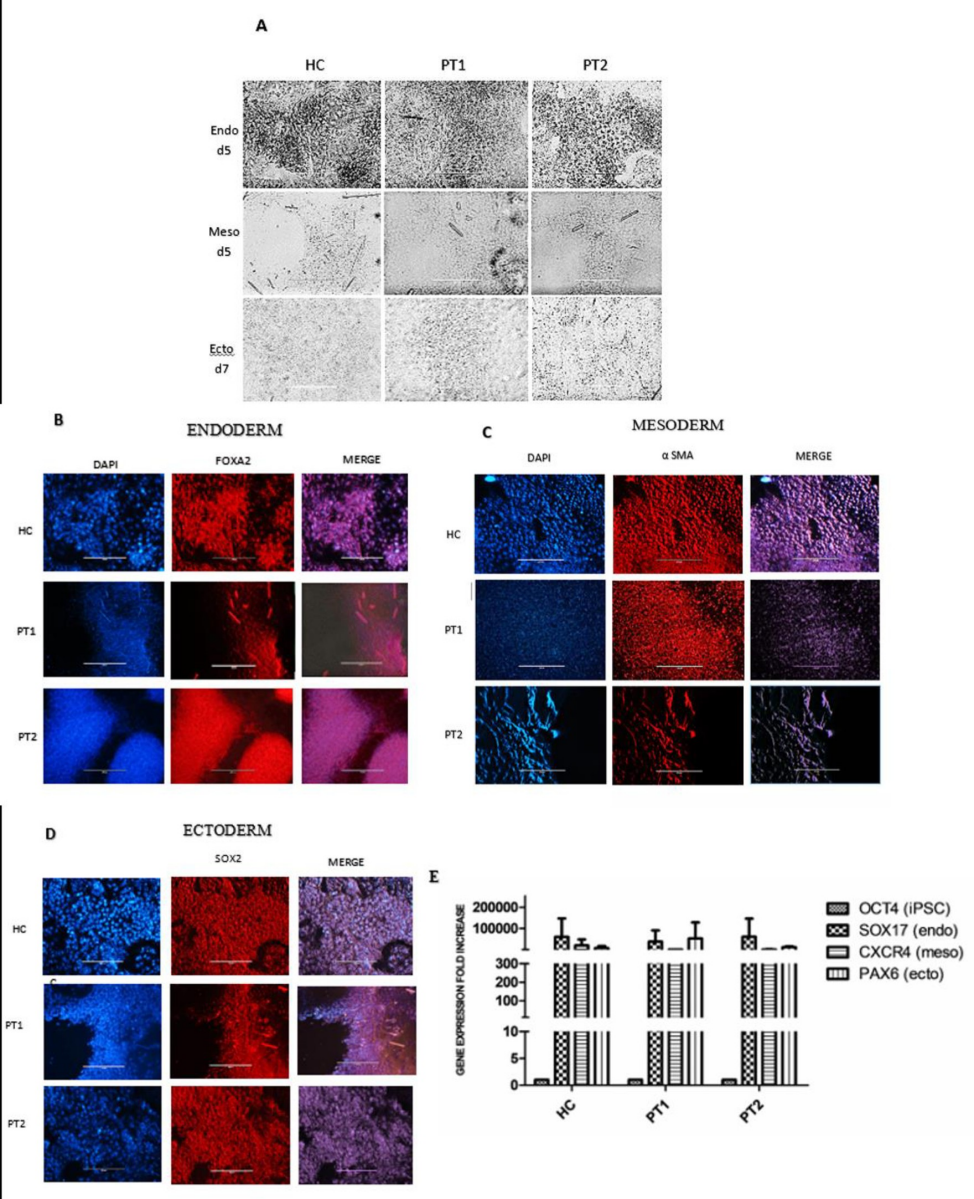
Characterization of the three germ layers. Healthy control (HC) and ADPKD1 patients (PT1 and PT2). A. Typical morphology of the three germ layers. Germ layers were characterized by immunofluorescence staining using specific antibodies. B. Endoderm (FOXA2), C. Mesoderm (α SMA) and D. Ectoderm (SOX2). Cell nuclei were stained with DAPI. The last columns show merged images. E. Gene expression of OCT4 indicates pluripotency of iPSCs, SOX17 was a marker for endoderm, CXCR4 was a marker for mesoderm and PAX6 was a marker for ectoderm (n = 2). OCT4 expression by iPSCs was used as an internal quantitative control. OCT4 expression was undetectable in cells of the three germ layers (scale: 200 μm).

iPSCs were further characterized by their ability to differentiate into the three germ layers. Typical images of the progression of germ layer development in cells from the HC are shown in S5 Fig. Development of the three germ layers was verified by typical morphology (Fig 2A) and by immunofluorescence labeling for differentiation markers, namely, FOXA2 for endoderm, smooth muscle actin (SMA) for mesoderm and SOX2 for ectoderm (Fig 2B–2D). Negative controls for the antibodies are shown in S7 Fig. Fig 2E shows the gene expression levels of specific markers of endoderm (SOX17), mesoderm (CXCR4) and ectoderm (PAX6), as detected by RT-PCR.

### Generation of kidney organoids from iPSCs

**Fig 3:**
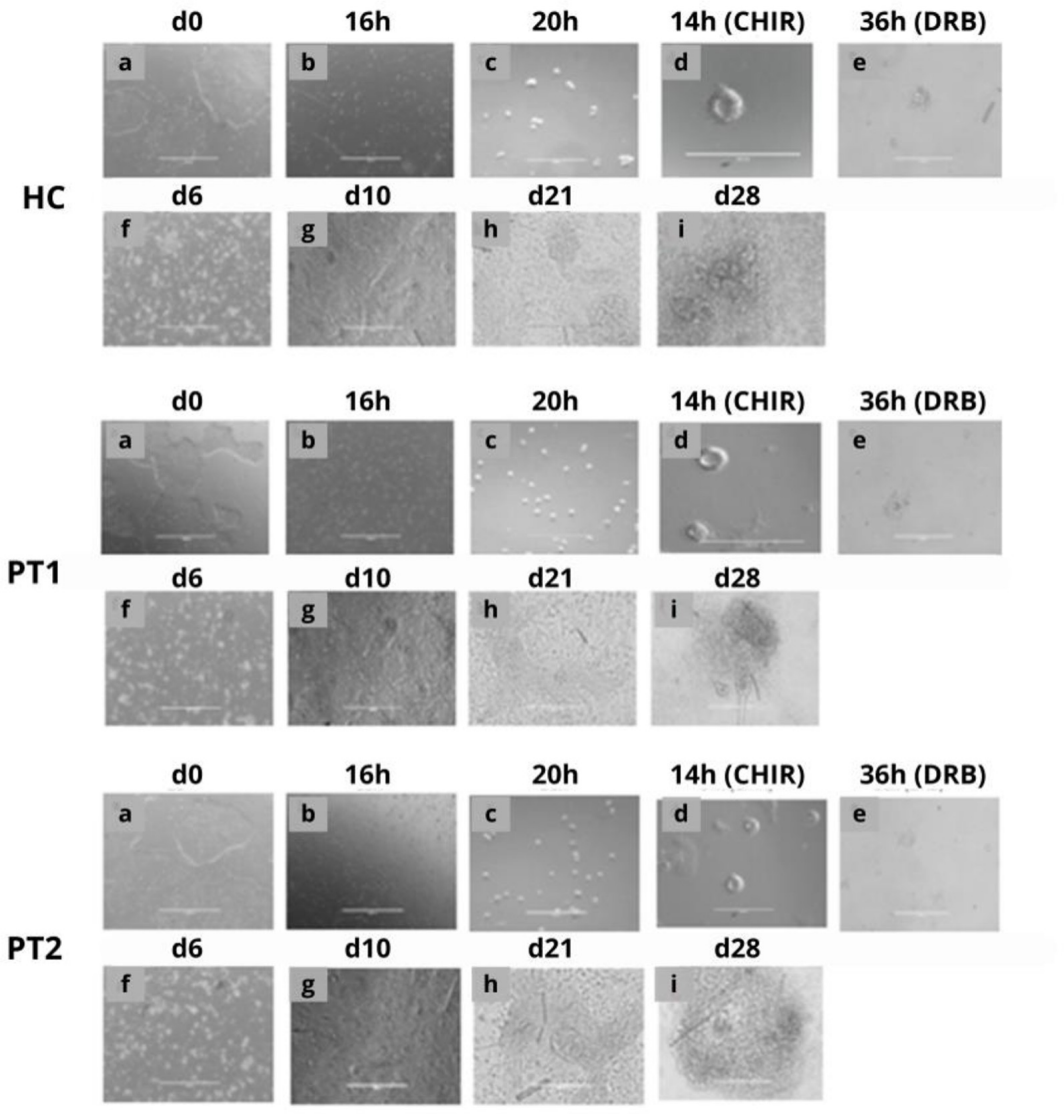
Generation of tubular organoids. Phase contrast images of the generation of epithelial tubular kidney organoids at 28 days from iPSCs generated from the HC, PT1 and PT2 groups. In the first 36 h, the iPSCs were maintained in 3D culture. Then, they were dissociated, transformed into single cells and treated with Y-27632, which allowed cellular reorganization and survival, producing epiblast spheroids (d). Then, CHIR99201 and DRB were added, activating the Wnt/β-catenin pathway and allowing differentiation into tubular organoids (i). The morphology among spheroids derived from ADPKD patients and from a HC donor is similar. (Scale bar for a and f: 1000μm; scale bar for b, c d, e, g, h and i: 200μm).

Three-dimensional renal structures were generated from iPSCs derived from the HC and both ADPKD patients following steps that recapitulate embryonic development. Fig 3 shows the steps that are necessary to obtain the respective embryonic structures. iPSC colonies (Fig 3A) were dissociated with Accutase (Fig 3B) to obtain single iPSCs (Fig 3C). Considering the normal embryogenic process, the colonies would be expected to differentiate into a blastula and then a gastrula, but the spontaneous differentiation of iPSCs in culture is still a challenge since cells spontaneously undergo apoptosis [16]. To circumvent this undesired outcome, a ROCK inhibitor of signaling (10 μM Rho-kinase inhibitor Y27632) was added 16 h after the single cell stage. Inhibition of the ROCK pathway prevents cells from triggering the death pathways, allowing them to survive and adjust to proceed towards the differentiation process. The iPSCs then grew until reaching the epiblast stage, which is characterized by the presence of spheroid cavities (Fig 3D). Next, a potent GSK-3b inhibitor/Wnt pathway agonist (CHIR99021) was added, which was followed by the addition of DRB medium to induce epithelial-mesenchymal transition (Fig 3E and 3F) and finally mesenchyme-epithelial transition (Fig 3G); these steps enabled epithelialization and sequential formation of pretubular aggregates and renal vesicles (Fig 3H), which ultimately enabled the formation of renal tubular organoids (Fig 3I). All these *in vitro* embryogenesis steps were similar for cells derived from both HC and ADPKD patients.

**Fig 4:**
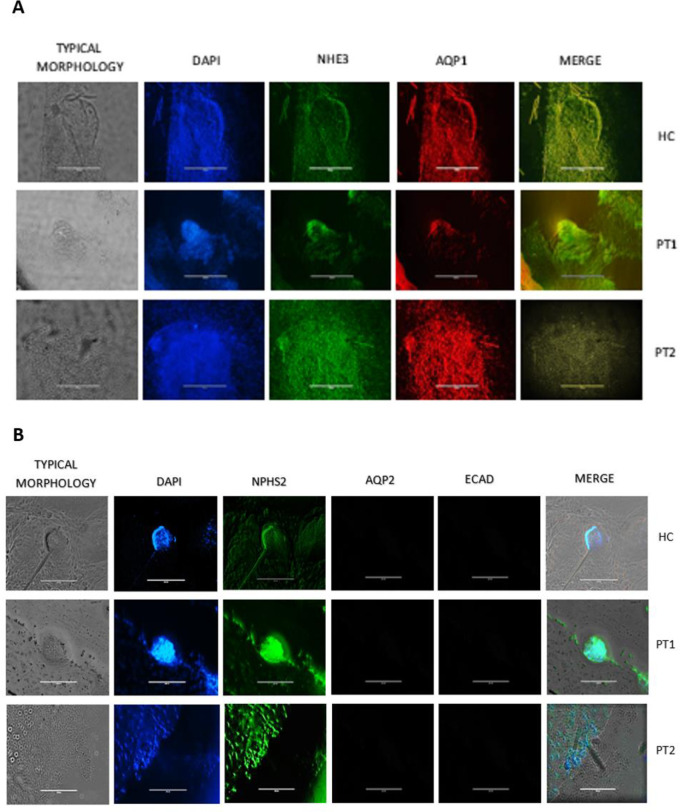
Kidney organoids. Representative immunostaining of epithelial tubular kidney organoids at 28 days for HC, PT1 and PT2 patients. Typical morphology of organoid structures (first columns). Cell nuclei were stained with DAPI (second columns A-B). A, each structure was characterized by immunofluorescence staining using specific antibodies against NHE3 (Abcam 1:500) and AQP1 (Sigma 1:500). B, NPHS2 (Abcam 1:100) was detected. AQP2 (Sigma 1:100) was not detectable, proving that only the metanephric mass of the structures was performed. ECAD (Sigma 1:100) was not detectable in the absence of forskolin. A-B: Images taken at 20X.(scale bar 200 μm).

The presence of tubular structures was verified by the expression of specific markers by immunofluorescence: NHE3 and AQP1 for proximal tubules (Fig 4A) and NPHS2 for glomeruli (Fig 4B). In contrast, AQP2 labeling was not detected (Fig 4B), confirming that this methodology allowed only the development of the metanephric mass but not of the ureteric bud. The marker ECAD was not detected in the absence of forskolin (Fig 4B).

**Fig 5:**
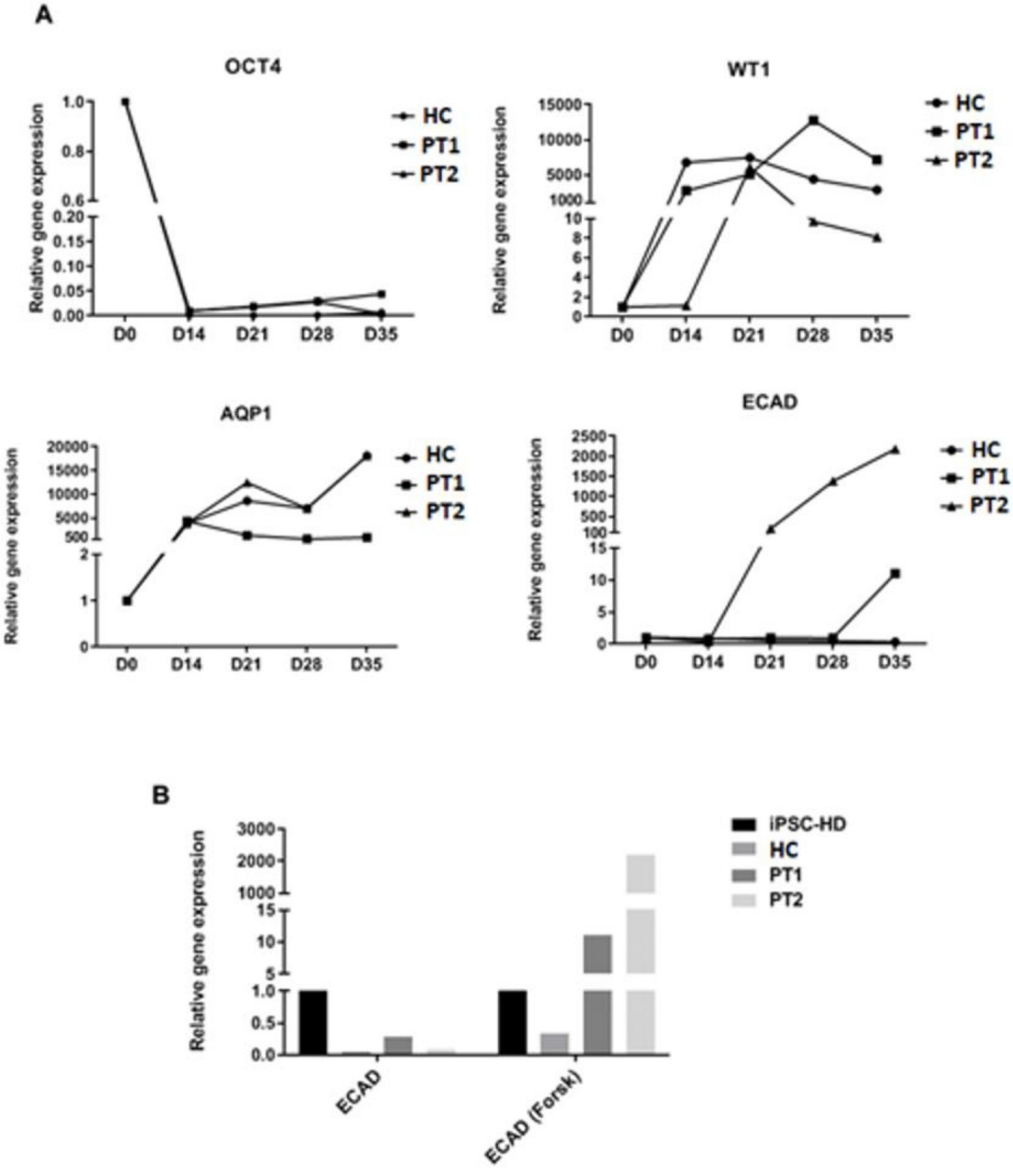
Relative gene expression levels of renal structures. A. Sequential expression of pluripotency and organogenesis markers. Day 0 (D0) refers to iPSCs and OCT4 expression in D0 was used as a quantitative standard for each sample. In this set of experiments, forskolin was added at day 28. B. Expression levels ECAD in the organoids at day 35 in the presence and absence of forskolin (added at day 28). GAPDH was used as an internal control and OCT4 expression by iPSCs from healthy donor (HD) was used as the quantitative standard. WT1 and AQP1 were used as markers of renal structures, and ECAD as the cyst marker.

To verify the presence of specific markers expressed throughout the process of progenitor kidney development, all samples (HC, PT1 and PT2) were collected at five points of the whole process of differentiation: day 0 (iPSCs), days 14, 21, 28 and then at day 35 (7 days after cyst induction). The time course of gene expression revealed differentiation during the embryogenesis process, as shown in Fig 5A–5C. The pluripotency marker OCT4 was expressed by iPSCs (day 0), but its levels were reduced over 14 days, reaching very low levels following differentiation. In contrast, the expression of the specific markers of metanephric mesenchyme (WT1) and proximal tubule (AQP1) progressively increased when the differentiation process was initiated by day 14, reaching a plateau by day 21. Fig 5D shows the expression of ECAD, a cystogenesis marker, at day 35, i.e. 7 days after cyst induction by forskolin. In the absence of forskolin, the expression of ECAD was very low, even in ADPKD patients. However, when forskolin was added (day 28), both patients exhibited increased ECAD expression; but the same pattern was not observed in HC organoid which remained with low levels of ECAD expression even in the presence of forskolin.

**Fig 6:**
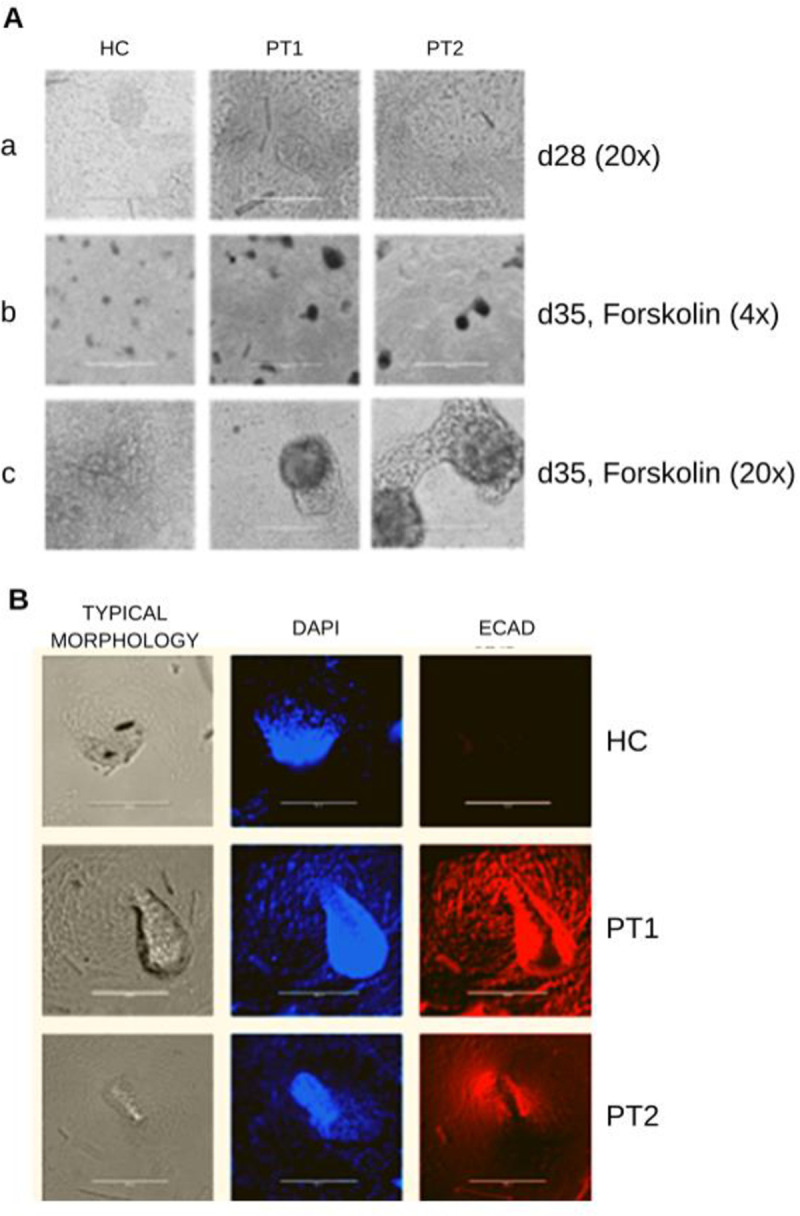
Organoid and cyst formation. Representative images of organoids after cyst induction with forskolin for HC, PT1 and PT2. Typical morphology of organoids on day 28 without forskolin induction (A-a). Images of several organoids on day 35 in culture upon forskolin induction 4X (A-b). Typical morphology on day 35 upon forskolin induction showing cyst formation, 20X (A-c). B Representative immunostaining of ECAD markers showing the typical morphology of kidney organoids. Cell nuclei were stained with DAPI (second columns). ECAD was not detected in the HC (R&D Systems 1:500), but it was detected in PT1 and PT2. A-B: 20X. (scale bar 200 μm).

The presence of cysts formed from the organoids stimulated with forskolin was evident for ADPKD patients, as shown in Fig 6. The upper panel shows images of the organoids at day 28, before the addition of forskolin (Fig 6Aa). Fig 6Ab (panoramic view-4X) and 6Ac (20X viewing) show the organoids at day 35 in the presence of forskolin showing the presence of cyst-like structures in samples from both patients (PT1 and PT2) but not in the HC sample. The presence of cysts was also verified by immunofluorescence staining (Fig 6B). ECAD labeling was not detected in the HC organoid, but it was clearly observed in the organoids from both patients. Interestingly, the presence of a cyst lumen can also be observed.

## Discussion

In the present study, we described in detail a procedure to generate kidney organoids from human iPSCs obtained from the blood erythroid progenitor cells of patients with ADPKD. Many somatic cell types have been successfully reprogrammed into iPSCs, including blood cells [7]. Here, we opted to use blood erythroid progenitors as the starting cell type for a number of reasons, such as their genomic integrity, epigenetic memory and efficient reprogramming [11, 17, 18]. The efficiency by which erythroid progenitors had been converted into iPSCs was as much as two orders of magnitude better (7%-28%) than the efficiency of differentiated blood cell types (0.02%-0.60%) or fibroblasts (0.74%) [7]. Despite the low abundance of these cells in the peripheral blood, they can easily be isolated and expanded *in vitro* to produce a sufficient number for reprogramming. One of the most important challenges in establishing suitable iPSCs is their genotype and phenotypic variability [6], but there are some technical strategies proposed to circumvent or reduce such variability. In the present experiments, we used a non-integrating episomal system for reprogramming that does not require genome integration for the reprogramming genes to be expressed [10], allowing less immunogenicity and rendering more genetically stable cells [19, 20]. Moreover, all samples were derived from the same source, which helps minimize epigenetic variability [12] assuming that differentiation of the starting cell might influence the efficiency of the end results. Analysis of the pluripotency markers and the ability of iPSCs to differentiate into all three germ layers were compared side-by-side, and they were also compared to the H9 embryonic cell line, which was used as a positive control. All these comparisons showed no significant differences between ADPKD patients, HCs and H9 cells.

Several protocols have been developed for inducing the differentiation of iPSCs into kidney organoids [13, 21]. Based on protocols described by Cruz et al. [6], we obtained structures with the characteristics of proximal tubules, including the expression of NHE3 and AQP1. In addition, the organoids expressed NPHS2, indicating the presence of podocyte-like cells. Conversely, AQP2 was not expressed in these tubular organoids. This finding is in agreement with previous studies pointing to several limitations in developing structures derived from the ureteric bud (UB) *in vitro*, with the development of UB requiring specific protocols, since the timing window for the addition of the specific inductor is very limited [6, 22, 23].

In the process of generating kidney organoids from iPSCs, all of renal embryogenesis is recapitulated *in vitro*, initially involving the formation of cavitated spheroids from single cells [14]. Indeed, we found that undifferentiated iPSCs from both ADPKD patients and healthy control formed epiblast-like spheroid structures that were morphologically similar. Although DNA sequencing of the PKD1 and PKD2 genes was not performed in the present study, the current findings indicate that the type of mutation carried by the patients had no influence on typical epiblast morphogenesis. Further studies are still needed to more properly evaluate the expression of pivotal molecules involved in this phase of embryonic development.

According to the protocol developed by Freedman et al. [14], we observed that spheroids did differentiate into tubular organoids 26 days after induction of differentiation, and the timing was similar for both ADPKD patients and HC (Fig 3). Nevertheless, we noticed some variability regarding the timetable of expression and the levels of differentiation markers. On the other hand, the morphology of the tubular structures was similar and comparable among samples from the ADPKD patients and HC. In contrast, using iPSCs originating from renal biopsies of ADPKD patients, Cruz et al, group found a higher variability in the ability of these cells to form organoids [6]. In addition to the huge diversity of mutations inherent to this disease, these differences in the efficiency of obtaining organoids may also be explained by the heterogeneous cell types present in biopsy samples, in contrast to the erythroid progenitors, which constitute a more homogeneous cell population. Taken together, these results indicate that despite the variability and differences in the efficiency of different iPSC clones to form kidney organoids, obtaining the latter from ADPKD patients may constitute a unique model for studying the multiple forms of phenotypical manifestations of this disease. Moreover, it is important to note that if there is a considerable variability of mutations in the ADPKD-affected population, the patient-specific iPSCs, being totally immune compatible, they might be used for individual regenerative therapies in the future, in addition to allowing the development of an *in vitro* model to verify whether the very early phenotypical manifestations are also as diverse as the clinical presentation.

Unstimulated organoids derived from HC or ADPKD patients did not spontaneously form cysts. The typical morphology of cysts and the expression of cystogenesis markers were not detectable in any sample, even extending the organoid differentiation in culture until completing 35 days. It has been demonstrated that cell proliferation alone does not produce cysts [24]. Moreover, a third independent event (known as third-hit) is required to accelerate cyst formation and growth *in vivo* [23]. Conversely, cyst formation induced by forskolin did occur in the organoids from ADPKD patients but not in those from HC. Of note, although present, cysts were observed at a low percentage in the ADPKD samples even in the presence of forskolin, as has also been observed by Freedman et al. [14] in organoids formed from the CRISPR/Cas9 model of ADPKD disease. Some hypotheses can be raised to explain the low frequency of cysts found in the present model. 1) Organoids cultured in 3D suspension milieu showed much higher cystogenesis efficiency, when compared with adherent organoids [6], indicating that the microenvironment is directly implicated in cystogenesis. 2) The experimental protocol employed in the present study formed proximal tubule organoids, but not distal portions of the nephron [23]. Although all nephron segments can originate cysts including proximal tubules [25], apparently those derived from collecting ducts appear to be larger and in higher numbers than those from other segments [26], 3) Finally, it was recently demonstrated that the origin of cysts may be related with the presence of multiple PKD1 mutations that determine the severity of the disease and that cyst formation in the collecting ducts was associated with disease severity [27, 28].

In conclusion, the present findings showed that it is feasible to generate organoids with three-dimensional structures similar to the kidney progenitor architecture using iPSCs reprogrammed from erythroid progenitors that were easily isolated from ADPKD patients and that these organoids are able to form cysts under forskolin stimulation. This promising methodology unveils a method for increasing understanding of the very early genetic, phenotypical and physiological steps in cyst formation in the ADPKD setting.

## Supporting information

S1 Fig Patients pedigree and MR images. (DOCX)

S2 Fig EP cells characterization (flow cytometry). (DOCX)

S3 Fig Karyotyping analysis of the iPSCs. (DOCX)

S4 Fig iPSC colonies. (DOCX)

S5 Fig Representative images of the three germ layers. (DOCX)

S6 Fig Molecular characterization of the germ layers. (DOCX)

S7 Fig Positive and negative controls for the antibodies. (DOCX)

S1 Table PCR primers sequence. (DOCX)

## Abbreviations

iPSCs: induced pluripotent stem cell
ADPKD: autosomal dominant polycystic kidney disease
EP: erythroid progenitors
HC: health control
PC-1: polycystic1
PC2: polycystic2
PDK: polycystic disease
GLYA: glycophorin A
qRT-PCR: quantitative real time PCR
HMBS: (hydroxymethylbilane synthase
GAPDH: gliceroldehyde 3 –phosphatase dehydrogenase
NPHS2: Nephrin2
AQP1: aquaporin1
AQP2: aquaporin2
NH3: NA+/H+exchanger isoform3
WT1: Wilms tumor
ECAD: E-cadherin
UB: ureteric bud
MM: metanephric mass
DNA: deoxyribonucleic acid
CRISPR/cas9: clustered regularly interspace short palindromic repeats
Oct4: octamer-binding transcription factor 4
SMA: smooth muscle actin
