# Supplementary figures and images for: Kidney organoids generated from erythroid progenitors cells of patients with autosomal dominant polycystic kidney disease
Source: PLoS One. 2021 Aug 2;16(8):e0252156. doi: 10.1371/journal.pone.0252156 (PMC8328284; doi:10.1371/journal.pone.0252156)

**
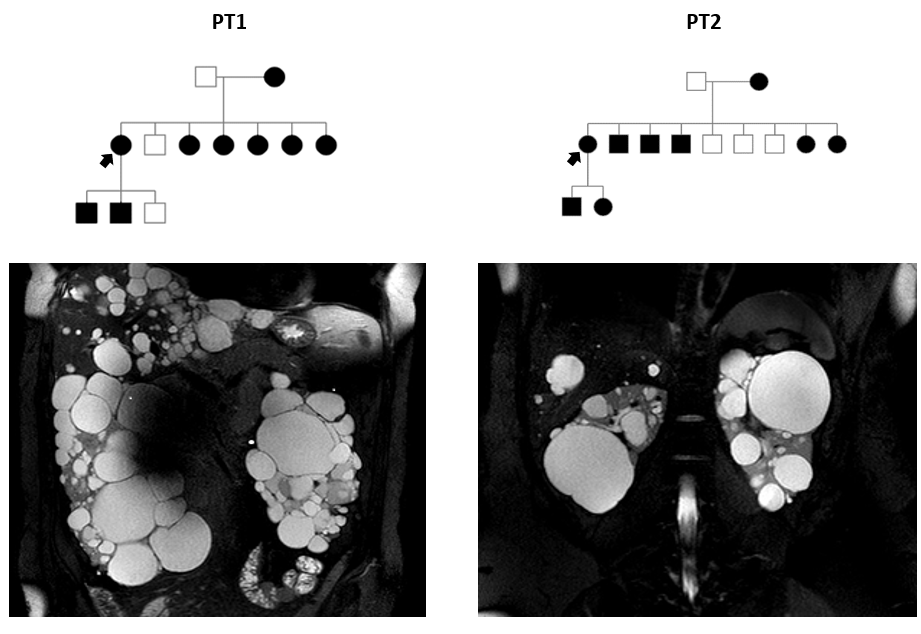
**

**S1 Fig -** Family pedigrees (A) and Magnetic Resonance Images (B) of both ADPKD patients (PT1 and PT2).

Supplement: S1 Fig — (DOCX) [file pone.0252156.s001.docx]
