# Supplementary material for: Kidney organoids generated from erythroid progenitors cells of patients with autosomal dominant polycystic kidney disease
Source: PLoS One. 2021 Aug 2;16(8):e0252156. doi: 10.1371/journal.pone.0252156 (PMC8328284; doi:10.1371/journal.pone.0252156)
Supplement: S2 Fig — (DOCX) [file pone.0252156.s002.docx]

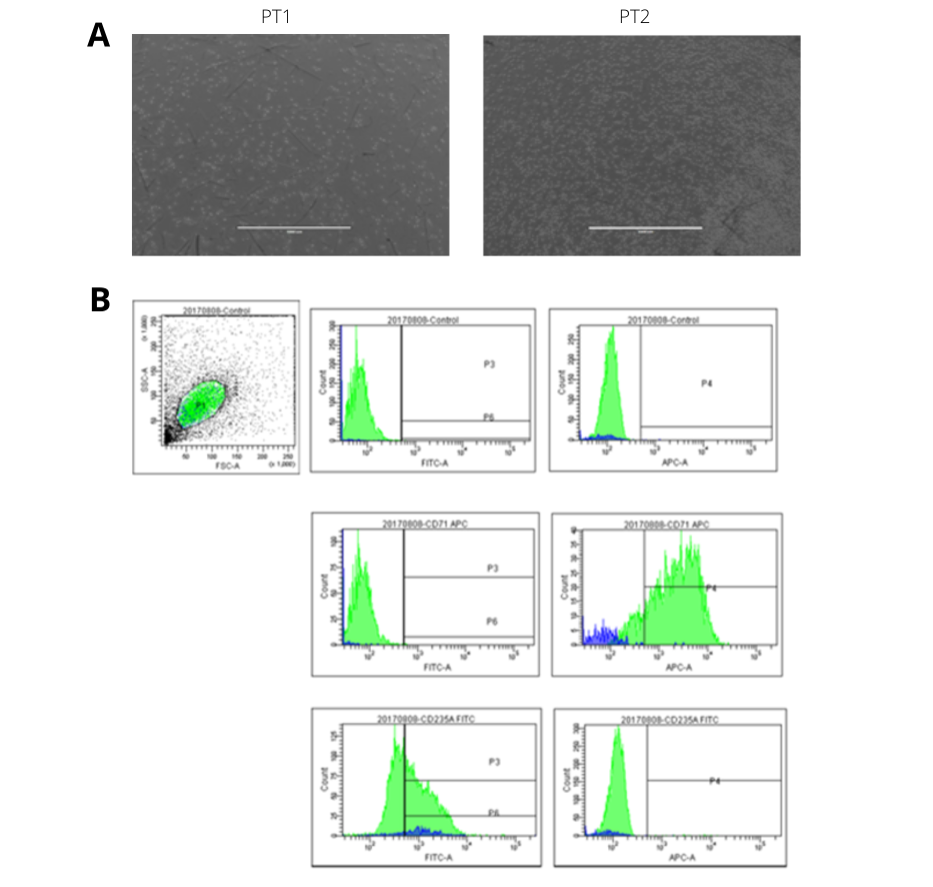


**S2 Fig**. Expansion and characterization of the progenitor erythroid cells (EP). A. Cell culture. EP were maintained during 48 days in specific expansion medium to reach a concentration of 1x10^6^ cells. B. Flow cytometry analysis. Upper panel: Negative marker. Middle panel: EP population were positive for CD71 (transferrin receptor) with 88% APC marker. Lower panel: EP population were positive for CD235A (Glycophorin A) with 57,4% FITC marker.
