# Supplementary material for: Kidney organoids generated from erythroid progenitors cells of patients with autosomal dominant polycystic kidney disease
Source: PLoS One. 2021 Aug 2;16(8):e0252156. doi: 10.1371/journal.pone.0252156 (PMC8328284; doi:10.1371/journal.pone.0252156)
Supplement: S3 Fig — (DOCX) [file pone.0252156.s003.docx]

**
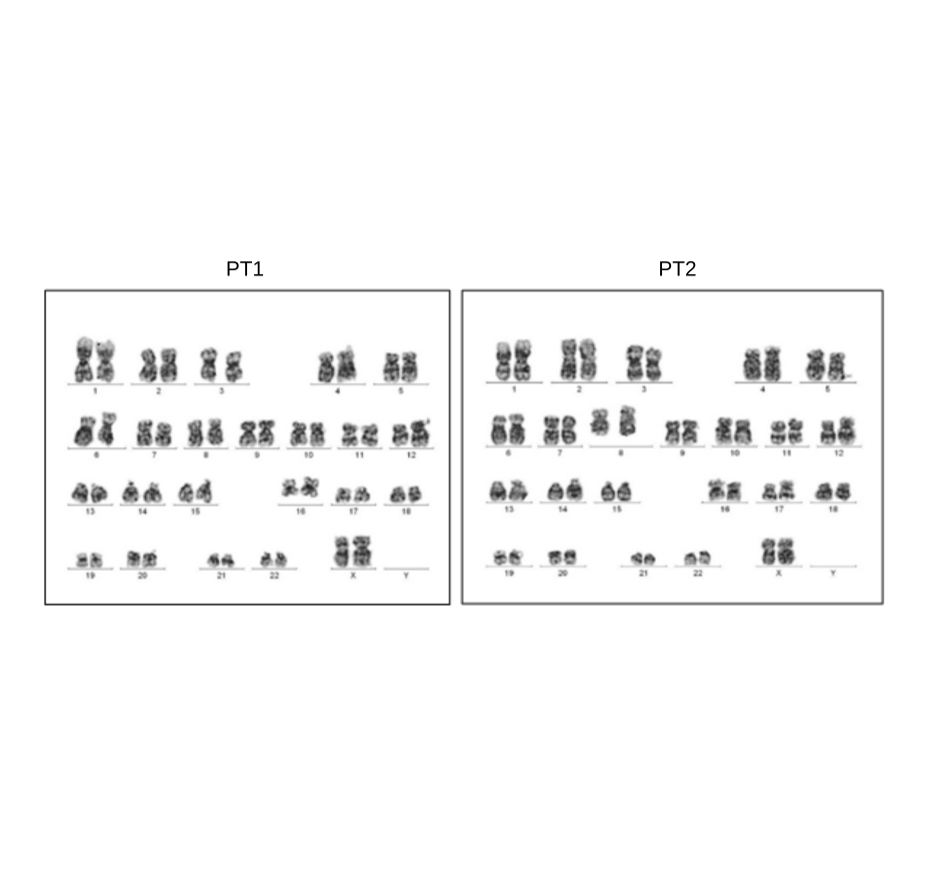
**

**S3 Fig**. G-banded karyotyping analysis. iPSCs showed perseveration of chromosomal integrity (46, XY) over the course of the reprogramming process in both of ADPKD patients.
