# Supplementary material for: Kidney organoids generated from erythroid progenitors cells of patients with autosomal dominant polycystic kidney disease
Source: PLoS One. 2021 Aug 2;16(8):e0252156. doi: 10.1371/journal.pone.0252156 (PMC8328284; doi:10.1371/journal.pone.0252156)
Supplement: S4 Fig — (DOCX) [file pone.0252156.s004.docx]

**
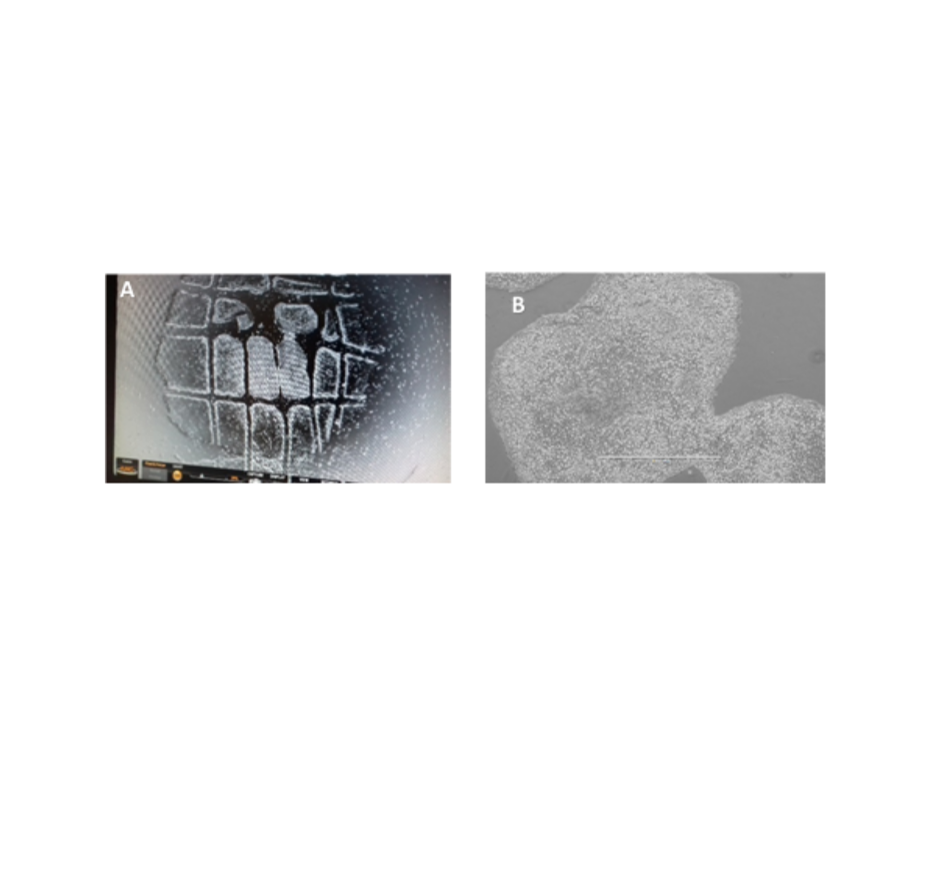
**

**S4 Fig.** A. The iPSC colonies were passaged manually by mechanical fragmentation, using a pipet tip under microscopy within the laminar flow. B. The colonies were dissociated by enzymatic digestion as recommendation by Gentle Cell (Life technologies) at 37^0^C for 7 minutes until edges of the colonies loose integrity and begin to lift from the dish.. Images taken using 20X magnification.
