# Supplementary material for: Kidney organoids generated from erythroid progenitors cells of patients with autosomal dominant polycystic kidney disease
Source: PLoS One. 2021 Aug 2;16(8):e0252156. doi: 10.1371/journal.pone.0252156 (PMC8328284; doi:10.1371/journal.pone.0252156)
Supplement: S5 Fig — (DOCX) [file pone.0252156.s005.docx]

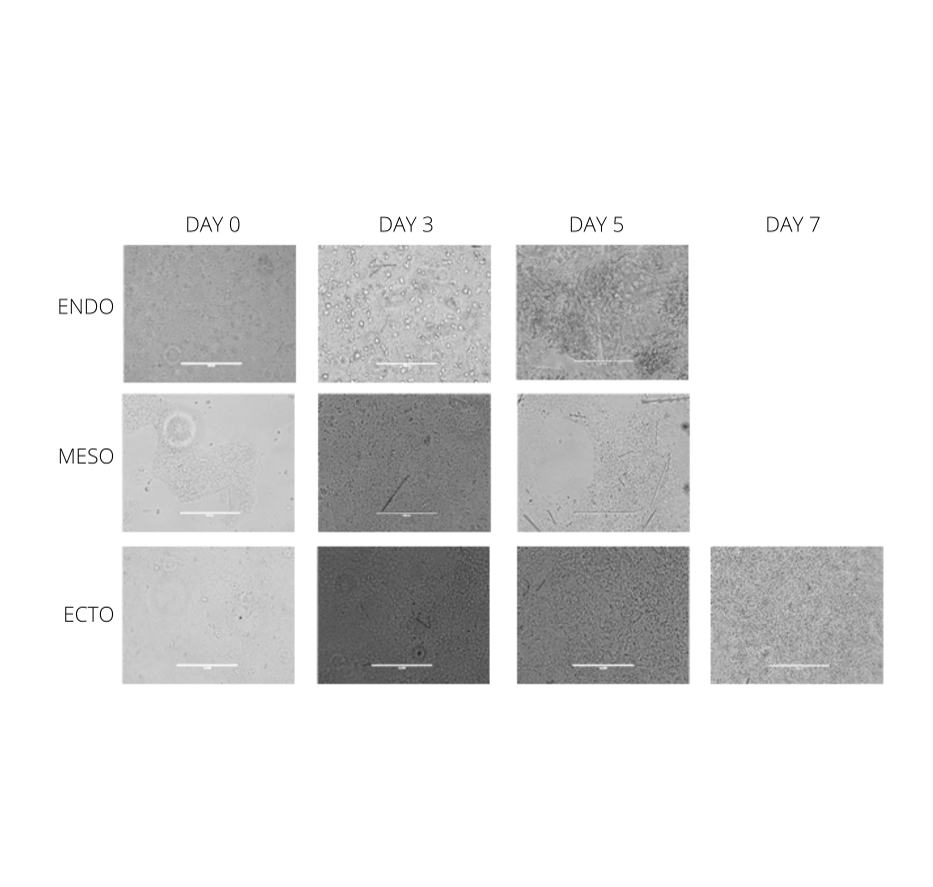


**S5 Fig**. Representative Images of the ability of iPSCs from the HC donor to differentiate into the three germ layers according STEMdiff Trilineage Differentiation protocol. Endoderm and mesoderm differentiation occurred over 5 days and ectoderm over 7 days.
