# Supplementary material for: Kidney organoids generated from erythroid progenitors cells of patients with autosomal dominant polycystic kidney disease
Source: PLoS One. 2021 Aug 2;16(8):e0252156. doi: 10.1371/journal.pone.0252156 (PMC8328284; doi:10.1371/journal.pone.0252156)
Supplement: S6 Fig — (DOCX) [file pone.0252156.s006.docx]

**
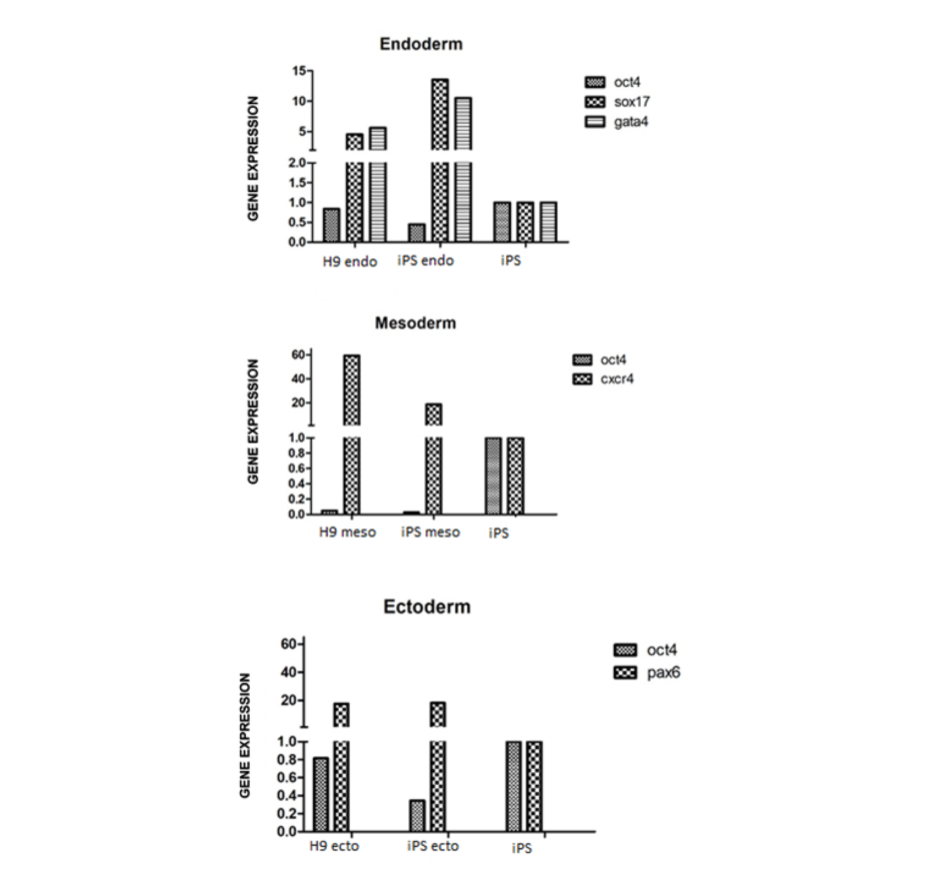
**

**S6 Fig.** Gene expression by RT-PCR of the markers of the 3 germ lineages from undifferentiated (iPSC) and differentiated iPSC (endo, meso and ecto) of the healthy control donor. H9 embryo cells were used as positive control. OCT4 was used as pluripotency marker. SOX7 and GATA4 was used for Endoderm, CXCR4 for Mesoderm and PAX6 for Ectoderm.
