# Supplementary material for: Kidney organoids generated from erythroid progenitors cells of patients with autosomal dominant polycystic kidney disease
Source: PLoS One. 2021 Aug 2;16(8):e0252156. doi: 10.1371/journal.pone.0252156 (PMC8328284; doi:10.1371/journal.pone.0252156)
Supplement: S7 Fig — (DOCX) [file pone.0252156.s007.docx]

**
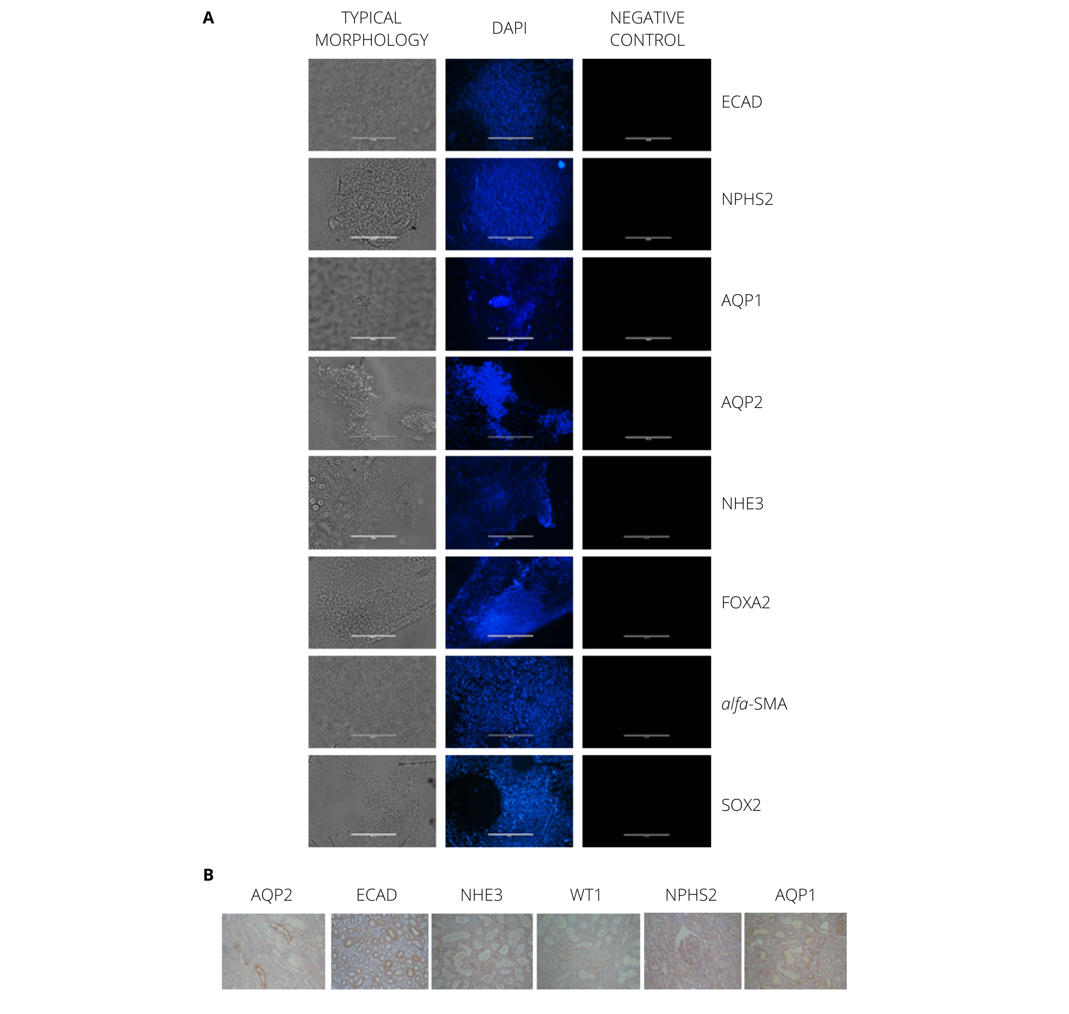
**

**S7 Fig**. Control analysis for the antibodies. A. Negative control. Primary antibodies were absent in IF reactions in kidney organoids. B. Human renal tissue was used as a positive control for antibodies by IHC.
