# Supplementary material for: Kidney organoids generated from erythroid progenitors cells of patients with autosomal dominant polycystic kidney disease
Source: PLoS One. 2021 Aug 2;16(8):e0252156. doi: 10.1371/journal.pone.0252156 (PMC8328284; doi:10.1371/journal.pone.0252156)
Supplement: S1 Table — (DOCX) [file pone.0252156.s008.docx]

| **S1 Table**. Primers sequence. Forward (F) and reverse (R). |  |  |  |  |
| --- | --- | --- | --- | --- |

| **GENE** | **PRIMER SEQUENCE** |
| --- | --- |
| AQP1 | F - GAAGTCGTAGATGAGTACAGCCAG  R - CTACACTGGCTGTGGGATTAACC |
| E-cadherin | F - TCGACACCCGATTCAAAGTG  R - GTCCCAGGCGTAGACCAAGA |
| GAPDH | F - GGTGGTCTCCTCTGACTTCAACA  R - ACCAGGAAATGAGCTTGACAAAG |
| WT1 | F - GGGTACGAGAGCGATAACCA  R - TCTCACCAGTGTGCTTCCTG |
| AQP2 | F - TACACCGGCTGCTCTATGAA  R - AGAAGACCCAGTGGTCATCAA |
| HMBS | F - TGGACCTGGTTGTTCACTCCTT  R - CAACAGCATCATGAGGGT TTTC |
| PAX6 | F - CACCGGTTTCCTCCTTCACA  R - TGGCAGAGCGCTGTAGGTGT |
| OCT4 | F - AAGCTCCTGAAGCAGAAGAGGA  R - ATGCTCGTTTGGCTGAATACCT |
| CXCR4 | F - CCCTGCCCTCCTGCTGACTATT  R - AGGGCCTTGCGCTTCTGGTG |
| SOX17 | F - CGCACGGACTTTGAACAATA  R - CAGACGTCGGGGTAGTTACAG |
